# Radiographic and anatomic landmarks to approach the anterior capsule in hip arthroscopy

**DOI:** 10.1093/jhps/hnv056

**Published:** 2015-09-01

**Authors:** Antonio Porthos Salas

**Affiliations:** 1. Hip Arthroscopy Mexico, San Pedro Garza, Garcia, Mexico; 2. Hip Arthroscopy Melbourne, Australia; 3. Swiss Hospital, Monterrey, Mexico.

## Abstract

Hip arthroscopy (HA) is considered to be a very difficult and demanding surgical procedure, special instruments, an image intensifier and a fracture table or hip distractor are required to access the hip joint, the most common and worldwide used HA technique is entering blindly to the central compartment with the use of fluoroscopy and continuous distraction; with the potential danger if performed in unskillful hands of labral penetrations, labral resections and scuffing of the femoral head cartilage. Our technique describes the arthroscopic management of femoroacetabular impingement (FAI), performing a preoperative planning using radiographic and anatomic landmarks to approach the anterior capsule without the use of fluoroscopy. Access to the hip joint is made extra-articularly from the peritrochanteric compartment palpating the greater trochanter and posteriorly penetrating the iliotibial band sliding the arthroscopic sheath and obturator from the trochanteric border to the anterior femoral neck to visualize the anterior capsule bursa and anterior capsule fibers and posteriorly following our previous landmarks perform an anterior oblique Inverted ‘T’ or ‘H’ capsulotomy with a radiofrequency wand to access the cam-type impingement and distraction is made under direct controlled arthroscopic vision. Our technique in HA aiming the anterior capsule using radiographic and anatomic landmarks is safe, reliable and reproducible in FAI with big cams, deep sockets and cases with mild arthritis where the capsule is thick, stiff and calcified.

## INTRODUCTION

Hip arthroscopy (HA) is considered to be a difficult and demanding surgical procedure to perform, its strong capsule, and deep constrained anatomy makes it very difficult to view and distract, the technique most used in HA is entering blindly the central compartment (CC) with fluoroscopy and continuous distraction; this technique has the danger if performed in unskillful hands of iatrogenic complications such as labrum penetrations, labrum resections and also scuffing of the femoral head cartilage [1, 2, 3].

New techniques have been developed to approach the hip joint extra-articularly and without fluoroscopy, others have a starting point from the peripheral compartment (PC) first [4, 5].

Our technique was developed due to the lack of appropriate material and instruments for HA at our institution, we approach the hip through the peritrochanteric compartment penetrating the iliotibial band (ITB) aiming towards the PC, sliding the arthroscopic sheath and obturator from the trochanteric border to the anterior femoral neck to visualize the anterior capsule bursa and anterior capsule fibre and posteriorly following our previous radiographic and anatomic landmarks perform an oblique anterior capsulotomy with a radiofrequency (RF) wand.

Knowledge of the hip joint anatomy is mandatory and a precise localization of the anterior capsule fibres is a must; the capsulotomy needs to be performed in an oblique direction to avoid hip instability [6].

## SURGICAL TECHNIQUE

**Fig. 1.**
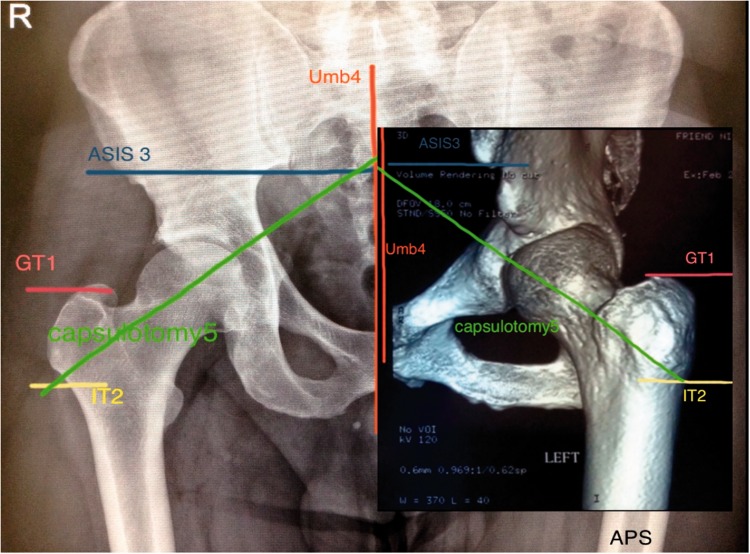
Radiographic landmarks plotted preoperatively in an AP of the Pelvis and also reproduced in an oblique view of a 3D CT scan.

**Fig. 2.**
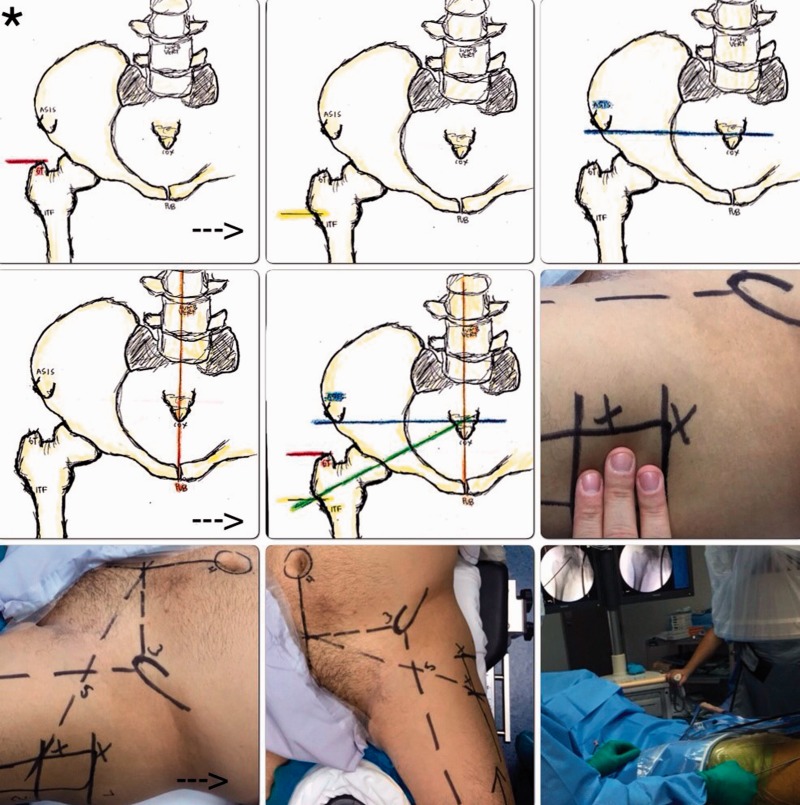
Preoperative planning key steps drawn one-by-one, observe how the intersection of the lines mimic the femoral neck axis (asterisk marks the starting point, arrow marks the direction of the pictures).

**Fig. 3.**
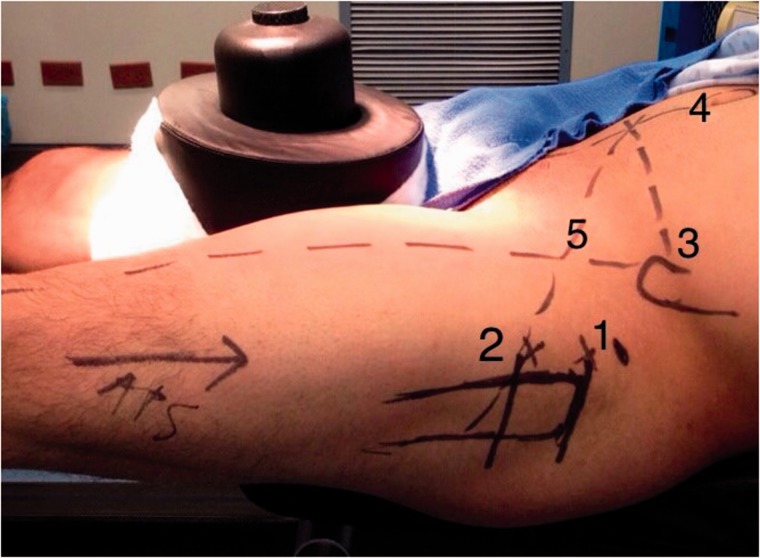
Landmarks reproduced in the patient’s operative left hip, observe the ***fifth line*** that exits the ***second line*** (innominate tuberosity of the femur) and mimics the femoral neck axis.

Patient setup in the modified supine position on a fracture table or distraction device, five radiographic lines or references are drawn preoperatively in an AP of the pelvis, where posteriorly are plotted in the patient’s operative hip (Figs. 1–3).

### Preoperative planning on an AP of the pelvis (Radiographic landmarks)

**Fig. 4.**
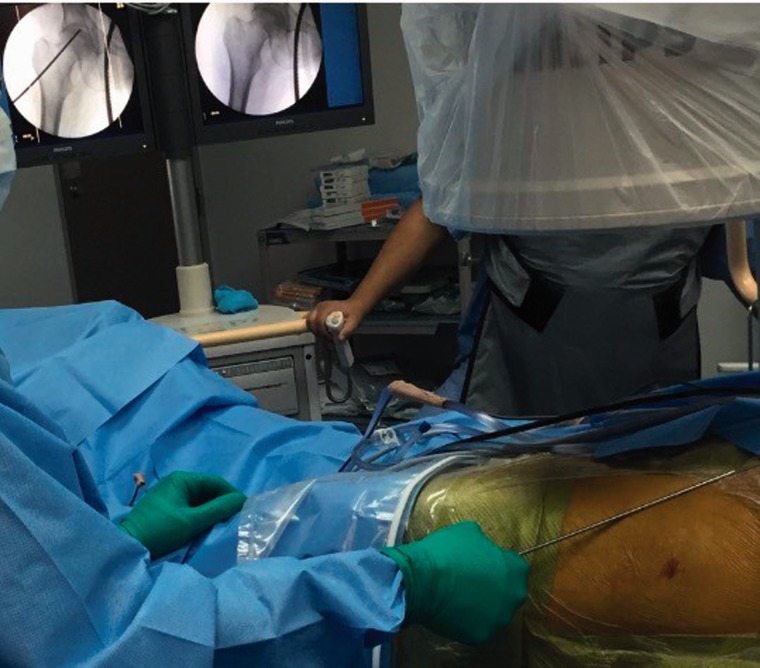
Intraoperative fluoroscopy test with a swithching stick to observe the desired position following the ***fifth or capsulotomy line*** (top left screen).

The *first line* is a horizontal line traced at the tip of the greater trochanter (GT), a *second horizontal line* is drawn at the innominate tuberosity of the femur, these two lines need to be measured with a standard ruler (generally they measure between 4 and 6 cm/3 finger breadths), *the third line*, is a horizontal line traced from the anterior superior iliac spine (ASIS) towards the vertebral body, *the fourth line* is a vertical line that runs from de superior mid vertebral body towards the pubic rami/pubic joint, the *fifth line* (capsulotomy line) is an oblique line rising or exiting from the innominate tuberosity of the femur to intersect the third and fourth line, this fifth line will closely represent and mimic the femoral neck axis and it is a guide to the surgeon to perform the anterior oblique capsulotomy (Fig. 4; Table I).

**Table I.** Capsulotomy steps plotted preoperatively on an AP of the pelvis

Localize the antero-superior border of the greater trochanter (GT) and trace a horizontal line, this corresponds to the ***FIRST LINE*.** Localize the innominate tuberosity of the femur (ITF) and trace a horizontal line, this corresponds to the ***SECOND LINE*.** Measure the distance between the first and second line, generally this distance measures 4–6 cm (3 finger breadths). Your ***THIRD LINE*** will run horizontally from the ASIS towards the lumbar vertebrae body or midline. Localize lumbar vertebral body and trace a vertical line towards the pubic rami or pubic joint, this corresponds to your ***FOURTH LINE*.** Localize the second line at the ITF and obliquely trace and intersect lines 3 and 4, this will correspond to your ***FIFTH LINE OR CAPSULOTOMY LINE*.**

^a^Key steps are represented in Fig. 2.

### Intraoperative planning on the patient’s hip (Anatomic landmarks)

Localize the tip of the GT and trace your first line, with three finger breadths (4–6 cm) from the GT distally trace you second line, palpate the ASIS and trace a line towards the midline on the patient’s belly, this corresponds to your third line, from the umbilical scar, trace your fourth line towards the pubic symphysis, the fifth line or capsulotomy line will exit from the innominate tuberosity of the femur (where the second line is placed) and it will intersect lines 3 and 4, this line mimics the femoral neck axis (Table II).

**Table II.** Capsulotomy steps plotted intraoperatively on the patient’s hip

Palpate and localize the greater trochanter (GT) and trace your ***FIRST LINE***, here you will place the AL or vision portal, slide the arthroscopic obturator and sheath penetrating the ITB towards the anterior capsule bursa and fibres. Use 3-finger breadths to place your ***SECOND LINE*** at the innominate tuberosity of the femur and place your PSP/working portal. Palpate the ASIS and trace a line towards the midline on the patient’s belly, this corresponds to your ***THIRD LINE****.* From the umbilical scar, trace your ***FOURTH LINE*** towards the pubic symphysis The **FIFTH LINE OR CAPSULOTOMY LINE** will exit from the innominate tuberosity of the femur (where the second line was placed) and it will intersect lines 3 and 4. Start your bursectomy with an RF wand or shaver, care must be taken to avoid bleeding. The marked ***FIFTH LINE*** is a guide to mimic the femoral neck axis and to perform a pristine anterior oblique capsulotmy.

^a^The plotted X-ray landmarks will be reproduced on the patient’s operative hip.

^b^Key steps are represented in Fig. 2.

**Fig. 5.**
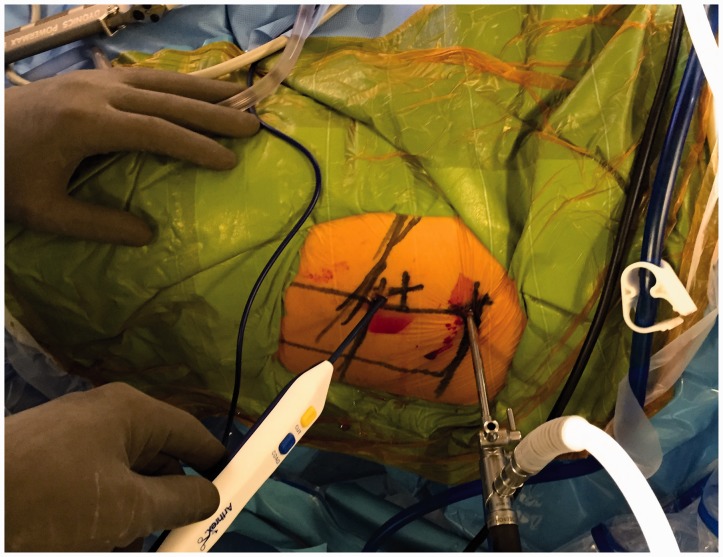
Portal placement on a left hip. AL used for vision located at the ***first plotted line***, PSP as a working portal and located at the ***plotted second line***. Observe how the RF wand follows the direction of the ***fifth plotted line*** to perform the capsulotomy.

**Fig. 6.**
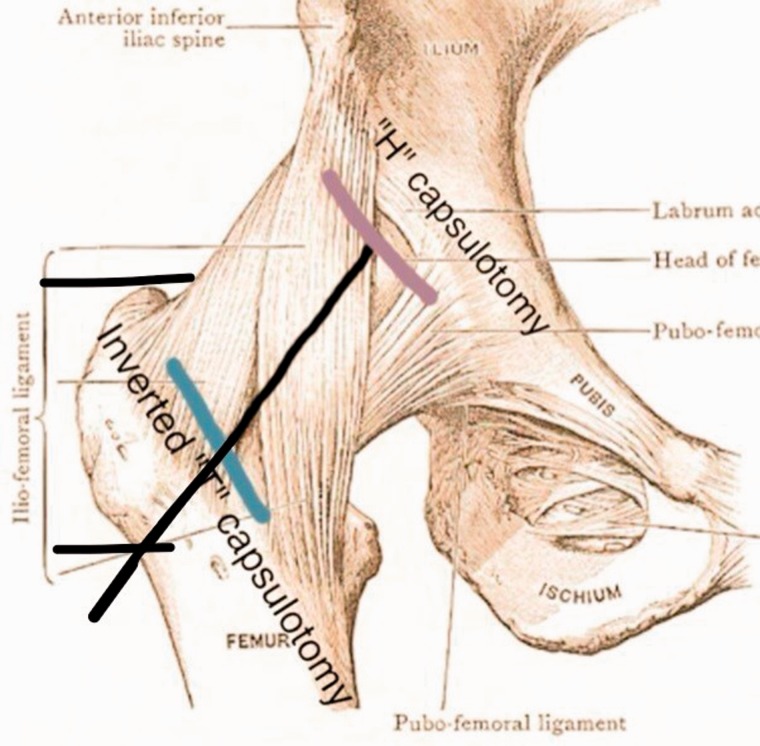
The anterior oblique ‘Inverted T’ or ‘H’ or capsulotomy.

**Fig. 7.**
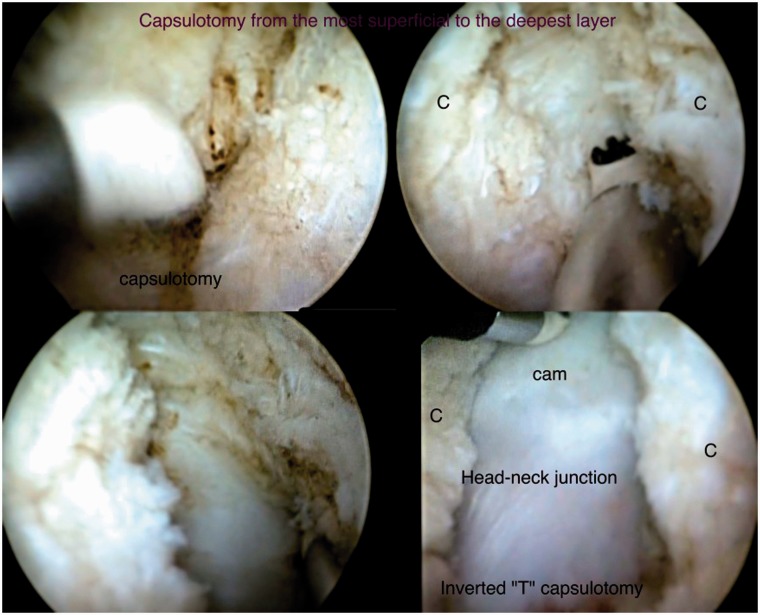
Capsulotomy performed from the acetabular rim (proximally) to the trochanteric crest (distally), observe the disection with the RF wand from the most superficial to the deepest layers of the capsule.

Portals are marked in the safe zone [6], the anterior margin of the GT is palpated, a stab incision is performed and the AL or vision portal is placed, posteriorly penetrating the ITB sliding the obturator and arthroscopic sheath to the anterior region of the femoral neck between the gluteus medius and iliocapsularis muscle, the PSP is placed at the level of the second line by triangulation, we localize the bursa, fatty tissue and the anterior capsule fibers, we perform a bursectomy with a RF wand or shaver, rotations are made to observe and assure how the capsule moves when the femoral head is rotating; we proceed with the anterior oblique ‘inverted T’ or ‘H’ capsulotomy (Figs. 5 and 6) proximally from the acetabular rim (taking care not to damage labrum) to the trochanteric crest distally, capsulotomy is performed with extreme precaution and care to avoid instability, the two arms of the capsule must be as close as possible to perform a 2–3-stich closure if necessary (Fig. 7), if we encounter with a cam femoroacetabular impingement (FAI), an ‘inverted T’-type capsulotomy is recommended to work only the head–neck junction, and if we encounter with a pincer FAI an ‘H’-type capsulotomy will suffice to work on the acetabular rim, sutures can be placed in both arms of the capsule to act as reins using your desire technique and device (Table III).

**Table III.** Tips and pearls

Hip rotations are mandatory to observe how the femoral head moves under the anterior capsule. Perform your bursectomy with an RF wand or shaver to observe the capsule fibres. Start your ‘Inverted T’ or ‘H’ capsulotomy superficially to the deepest layers of the capsule. Run your capsulotomy proximally from the acetabular rim to the trochanteric crest distally to increase visualization of the neck, head–neck junction, femoral head and extraarticularly the labrum. Care must be taken to avoid iatrogenic damage of the labrum with your RF wand. Start the capsulotomy in extension and posteriorly in slight flexion, abduction and external rotation of the hip. If difficulty is encounter in performing the capsulotomy, feel confident to take from 1 to 3 shots of fluoroscopy to observe the femoral neck axis. Distraction of the hip to enter the CC is made under direct arthroscopic vision after finishing with your FOC. Exchange to a 70° scope can be done entering the CC with switching sticks or your desire technique. Closure of the capsule must be done in patients with generalized ligamentous laxity to avoid instability of the hip.

Exchange to a 70° scope is done and distraction of the hip joint is performed with controlled arthroscopic vision (Fig. 7) where meticulous visualization and inspection of chondral–labral junction, labrum, acetabular and femoral head cartilage as well as the ligamentum teres.

**Fig. 8.**
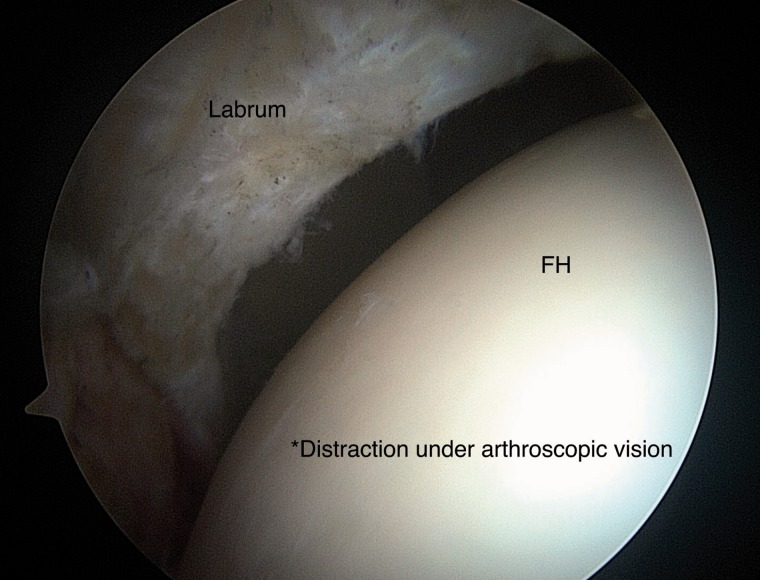
Distraction of the hip joint to enter the CC is prerformed under controlled direct arthroscopic vision.

Finishing in the CC, we remove the traction half way to infiltrate viscosupplementation (previous suck drying of saline) and removal of all traction is done via arthroscopic direct vision (Fig. 8).

## MATERIAL AND METHODS

Eighty-seven cases where included in this study, 59 right hips, 28 left hips, 46 cam-type FAI, 25 cases with mixt-type FAI, 4 with type 3 OA and mixt FAI, 4 osteonecrosis of the femoral head, 3 ligamentum teres tears (LTT) 2 hip dysplasia, 1 revision HA for adhesions, 1 case of a rigid hip due to lipomeningocele, 1 case of rheumatoid arthritis with type 4 brooke heterotopic bone formation.

Twelve patients with take down and labral refixation, 8 rim recessions, 85 had femoral osteochondroplasty, 4 patients with retrograde and arthroscopic guidance core decompression (CD), 1 case with removal of heterotopic bone, 3 patients with LT debridement.

Traction times (TT) were recorded, being the shortest one 18 min in a LT tear debridement case and the longest TT was 80 min in a core decompression of the femoral head (CDFH) case, patients with hip dysplasia had a 2-stitch capsular closure, 1 patient who had a labral refixation presented penis numbness for 7 weeks and 1 patient who had a HA and CDFH presented erectile dysfunction for 5 weeks, 3 patients were sent to physical therapy and started to early with hip extensions and presented intra-articular hematoma who needed extraction.

We did not encounter with infections, nor hip instability.

## DISCUSSION

Hip Preservation surgery and HA is a popular surgical procedure nowadays because of its excellent results, the knowledge of FAI is now being international thanks to worldwide meetings, cadaver labs and master courses, but the concern and worry of HA being performed by an undertrained surgeon with unskillful hands is major, due to the fact that the procedure itself is demanding, the use of fluoroscopy is needed to avoid devastating iatrogenic complications, so knowing the anatomy of the hip joint is an extremely important factor.

Thaunat *et al.* [7] mention that less force of traction is applied when the capsulotomy is done prior distraction and also that distraction is made only for accessing the CC or acetabular fossa, their results demonstrated that soft tissue injuries and nerve dysfunction are extremely rare, Doron et al. [8] describe an extracapsular technique for the non-distractable hip, but they still use fluoroscopy to establish the AL portal and also they perform a trial of distraction previous the capsulotomy is done.

With this technique, like the other published extracapsular ones, we have encounter with less iatrogenic complications such as scuffing of the femoral head, labral penetrations and resections, the risk of radiation to the patient, the theatre staff and the surgeon is cero or extremely low (first 3 cases when starting the technique) [9].

We believe that our technique performed with radiographic preoperative landmarks will mimic as close as possible the femoral neck axis, which is a guide to perform a pristine anterior oblique capsulotomy to permit a more natural capsule closure (longitudinal capsule fibres) in patients who do not need capsule closure, in patients with generalized ligamentous laxity the two arms of the capsule must be as close as possible to perform a 2–3-stitch closure, we strongly believe that this technique is surgeon friendly, very easy to perform, master, teach and there is no need for special cannulated obturators, guide wires or fluoroscopy.

Our technique in HA aiming the anterior capsule using radiographic and anatomic landmarks is safe, reliable and reproducible in FAI with big cams, deep sockets and cases with mild arthritis where the capsule is thick, stiff and calcified.

## CONFLICT OF INTEREST STATEMENT

None declared.
